# HIV silencing and cell survival signatures in infected T cell reservoirs

**DOI:** 10.1038/s41586-022-05556-6

**Published:** 2023-01-04

**Authors:** Iain C. Clark, Prakriti Mudvari, Shravan Thaploo, Samuel Smith, Mohammad Abu-Laban, Mehdi Hamouda, Marc Theberge, Sakshi Shah, Sung Hee Ko, Liliana Pérez, Daniel G. Bunis, James S. Lee, Divya Kilam, Saami Zakaria, Sally Choi, Samuel Darko, Amy R. Henry, Michael A. Wheeler, Rebecca Hoh, Salwan Butrus, Steven G. Deeks, Francisco J. Quintana, Daniel C. Douek, Adam R. Abate, Eli A. Boritz

**Affiliations:** 1 Department of Bioengineering and Therapeutic Sciences, School of Pharmacy, University of California, San Francisco, San Francisco, CA USA; 2 Ann Romney Center for Neurologic Diseases, Brigham and Women’s Hospital, Harvard Medical School, Boston, MA USA; 3 Department of Bioengineering, California Institute for Quantitative Biosciences, QB3, University of California, Berkeley, Berkeley, CA USA; 4 Virus Persistence and Dynamics Section, Vaccine Research Center, National Institute of Allergy and Infectious Diseases, National Institutes of Health, Bethesda, MD USA; 5 Human Immunology Section, Vaccine Research Center, National Institute of Allergy and Infectious Diseases, National Institutes of Health, Bethesda, MD USA; 6 Broad Institute of MIT and Harvard, Cambridge, MA USA; 7 Department of Medicine, University of California, San Francisco, San Francisco, CA USA; 8 Department of Chemical and Biomolecular Engineering, California Institute for Quantitative Biosciences, QB3, University of California, Berkeley, Berkeley, CA USA

**Keywords:** Pathogens, HIV infections, HIV infections

## Abstract

Rare CD4 T cells that contain HIV under antiretroviral therapy represent an important barrier to HIV cure^1–3^, but the infeasibility of isolating and characterizing these cells in their natural state has led to uncertainty about whether they possess distinctive attributes that HIV cure-directed therapies might exploit. Here we address this challenge using a microfluidic technology that isolates the transcriptomes of HIV-infected cells based solely on the detection of HIV DNA. HIV-DNA^+^ memory CD4 T cells in the blood from people receiving antiretroviral therapy showed inhibition of six transcriptomic pathways, including death receptor signalling, necroptosis signalling and antiproliferative Gα12/13 signalling. Moreover, two groups of genes identified by network co-expression analysis were significantly associated with HIV-DNA^+^ cells. These genes (*n* = 145) accounted for just 0.81% of the measured transcriptome and included negative regulators of HIV transcription that were higher in HIV-DNA^+^ cells, positive regulators of HIV transcription that were lower in HIV-DNA^+^ cells, and other genes involved in RNA processing, negative regulation of mRNA translation, and regulation of cell state and fate. These findings reveal that HIV-infected memory CD4 T cells under antiretroviral therapy are a distinctive population with host gene expression patterns that favour HIV silencing, cell survival and cell proliferation, with important implications for the development of HIV cure strategies.

## Abstract (web summary)

HIV-infected memory CD4 T cells under antiretroviral therapy are a distinctive population of cells with transcriptomic patterns that favour HIV silencing, cell survival and cell proliferation.

## Main

Understanding how HIV persists during antiretroviral therapy (ART) can advance the search for a safe and scalable HIV cure. A central example of this is the latent reservoir concept, in which some HIV proviruses are thought to persist by maintaining a quiescent state that spares their host cells from virus- or immune-mediated killing^2^. Evidence supporting this concept includes the presence of rare memory CD4 T cells in ex vivo samples that inducibly express HIV^1,3,4^, as well as data from culture models demonstrating molecular blocks to HIV transcription, particularly in resting cells^5–11^. These and other findings have prompted the development of latency-reversing agents (LRAs) that can induce HIV transcription with the goal of exposing infected cells to elimination in vivo. However, the lack of a demonstrable reduction in reservoir size in clinical trials of LRAs^12–16^ has emphasized how much remains unknown about the barriers to an HIV cure. Of particular importance is the long-standing uncertainty about the biology of HIV-infected CD4 T cell reservoirs. As cells containing quiescent viruses in the blood and tissues have not been identifiable without substantial manipulation, it has been impossible to establish whether these rare cells have special attributes that favour HIV latency or otherwise help to account for HIV persistence under ART. Studies attempting to circumvent this obstacle by detecting HIV enrichment in phenotypic, functional or anatomic CD4 T cell subsets^17–27^—in some cases using advanced single-cell analyses^28,29^—have found low levels of infected cells across subsets and emphasized the heterogeneity of the infected cell pool. Thus, the identification of distinctive biological signatures among HIV-infected CD4 T cells under ART has emerged as a central challenge in HIV cure research.

To help address this challenge, we developed a custom microfluidic technology that enables the unbiased detection and gene expression profiling of HIV-infected cells directly ex vivo. The technology, termed focused interrogation of cells by nucleic acid detection and sequencing (FIND-seq)^30^, separates millions of single cells within water-in-oil droplets for immediate lysis, followed by polyadenylated RNA sequence recovery and then sorting according to HIV DNA detection. This approach isolates whole transcriptomes from cells containing quiescent viruses without the need for in vitro latency reversal, thereby capturing a transcriptome-wide profile of these cells in their natural state. Here we used FIND-seq in people with HIV receiving long-term ART to analyse host gene expression patterns of memory CD4 T cells containing HIV *gag* DNA—a marker of the HIV-infected cell reservoir that encompasses both intact and defective virus sequences^31^. Our results reveal distinctive transcriptomic signatures that help to explain HIV-infected CD4 T cell persistence despite the suppression of virus replication, highlighting important opportunities for further progress towards an HIV cure.

## HIV-DNA^+^ cell transcriptome sorting

**Fig. 1:**
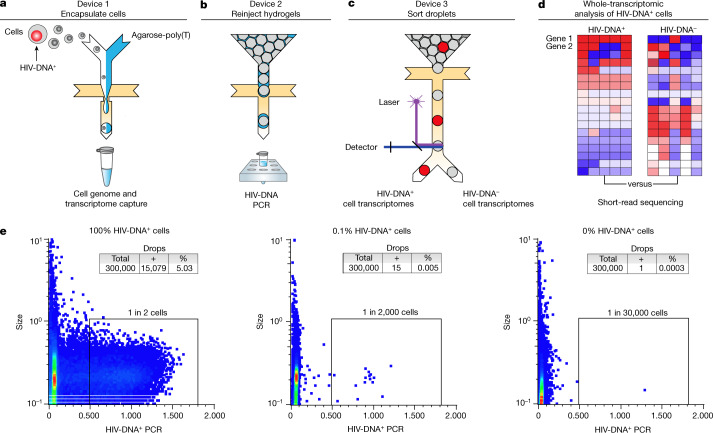
Whole-transcriptomic analysis of HIV-DNA^+^ cells using FIND-seq. **a**–**d**, Overview of the workflow, including three sequential microfluidic devices separated by handling steps. **a**, On the first device, single cells are encapsulated at a limiting dilution in a water-in-oil emulsion with lysis buffer and molten agarose-poly(T). The agarose is then cooled to form a hydrogel bead that retains genomic DNA and polyadenylated RNA. After oil removal, whole-transcriptome cDNA is covalently linked to the hydrogel by reverse transcription for subsequent whole-transcriptome amplification (WTA) using PCR (see Extended Data Fig. 1a–e). **b**,**c**, Hydrogel beads re-encapsulated on the second device are analysed using droplet PCR for HIV *gag* (**b**) and then sorted on the third device (**c**). **d**, The processing steps performed after droplet sorting include WTA, library preparation and sequencing, and bioinformatic comparison of HIV-DNA^+^ cells and HIV-DNA^−^ cells. **e**, Droplet cytometry plots demonstrating the analysis of pure HIV-DNA^+^ J-Lat T cells (left), pure HIV-DNA^−^ Jurkat T cells (right), and a mixture of 0.1% J-Lat and 99.9% Jurkat cells (middle). Cells were encapsulated at 1 cell per 10 droplets.

**Extended Data Fig. 1:**
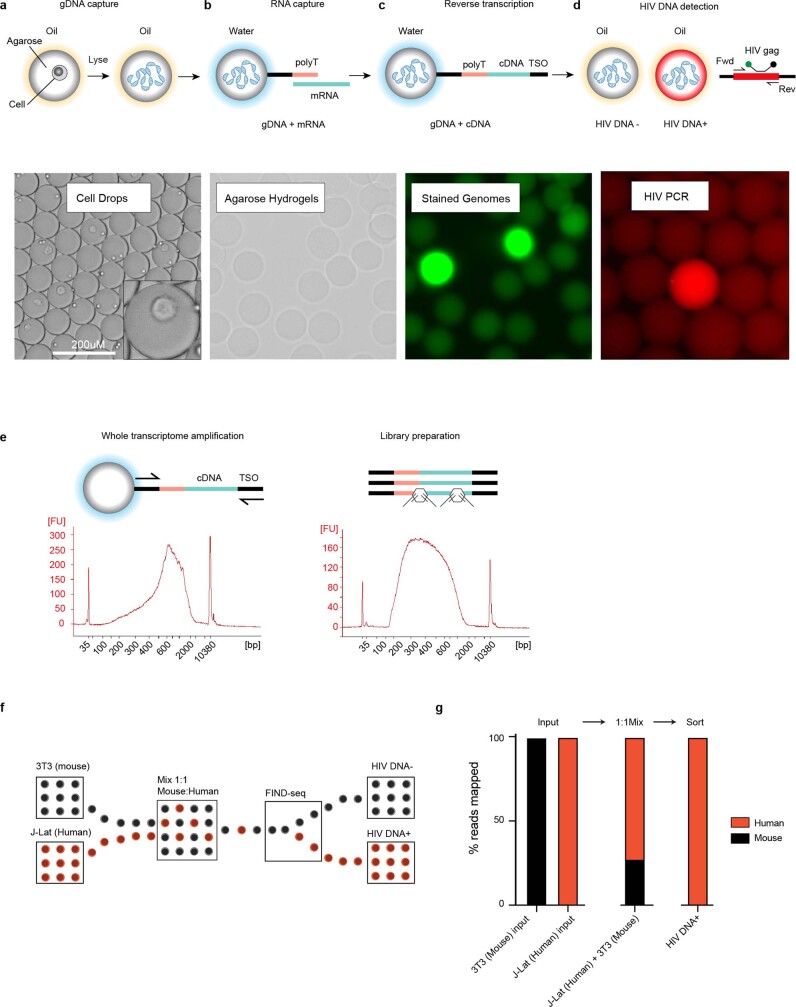
FIND-seq workflow details and sorted transcriptome purity. (**a**–**d**) Transcriptome recovery and HIV *gag* DNA detection steps in FIND-seq, including (**a**,**b**) capture of single-cell genomes and transcriptomes in agarose, (**c**) reverse-transcription of polyadenylated RNA in agarose following oil removal and washes, generating single-cell agarose hydrogel beads with retained genomic DNA and covalently linked whole transcriptome cDNA that incorporates a template-switch oligonucleotide (TSO) for subsequent WTA, and (**d**) HIV detection PCR using *gag* primers and hydrolysis probe after hydrogel re-encapsulation on Device 2 (Fig. 1). (**e**) Diagram of whole transcriptome amplification and library preparation from material sorted by FIND-seq, with representative microelectrophoresis traces. (**f**,**g**) Purity of transcriptome material sorted by FIND-seq. (**f**) HIV DNA^+^ J-Lat human T cells and HIV DNA^−^ 3T3 mouse fibroblasts were mixed and subjected to FIND-seq followed by whole transcriptome sequencing. (**g**) Percentages of transcriptome reads from pure input cell populations, the 1:1 mixture of 3T3 and J-Lat, and HIV DNA^+^ cell transcriptomes sorted by FIND-seq that aligned unambiguously to human (red) or mouse (black) references. Results from a single experiment are shown.

FIND-seq uses three microfluidic devices to isolate polyadenylated RNA sequences from HIV-DNA^+^ cells (Fig. 1a–c). The first device loads millions of single cells into water-in-oil droplets with a strongly denaturing lysis buffer and molten agarose covalently conjugated to oligo-dT (Fig. 1a). After encapsulation, the agarose in each single-cell droplet is cooled to form a hydrogel that retains high-molecular-mass DNA as well as polyadenylated RNA. This approach maintains compartmentalization among cells during oil removal, incubations, washes and reagent exchanges, therefore enabling optimized cell lysis, mRNA reverse-transcription and subsequent PCR while preventing interference between steps (Extended Data Fig. 1a–d). The second device reinjects washed hydrogels containing single-cell transcriptome cDNA and genomic DNA into a second emulsion for HIV *gag* DNA detection (Fig. 1b). The third device uses an accurate dielectrophoretic sorter^32^ to separate droplets on the basis of their fluorescence (Fig. 1c) for subsequent whole-transcriptomic analysis (Fig. 1d and Extended Data Fig. 1e). Using dilutions of latently infected human J-Lat T cells in uninfected human Jurkat T cells, FIND-seq droplet cytometry detected HIV-DNA^+^ cells with an estimated sensitivity of 50% and a per-droplet false-positive rate of 1 in 300,000 (Fig. 1e). Transcriptome sequencing in HIV-DNA^+^ droplets sorted from a 1:1 mixture of J-Lat and mouse cells revealed >99% human sequences (Extended Data Fig. 1f,g). These findings demonstrate that FIND-seq accurately detects rare HIV-DNA^+^ cells and isolates the transcriptomes from these cells.

## Transcriptome sequencing after FIND-seq

**Extended Data Fig. 2:**
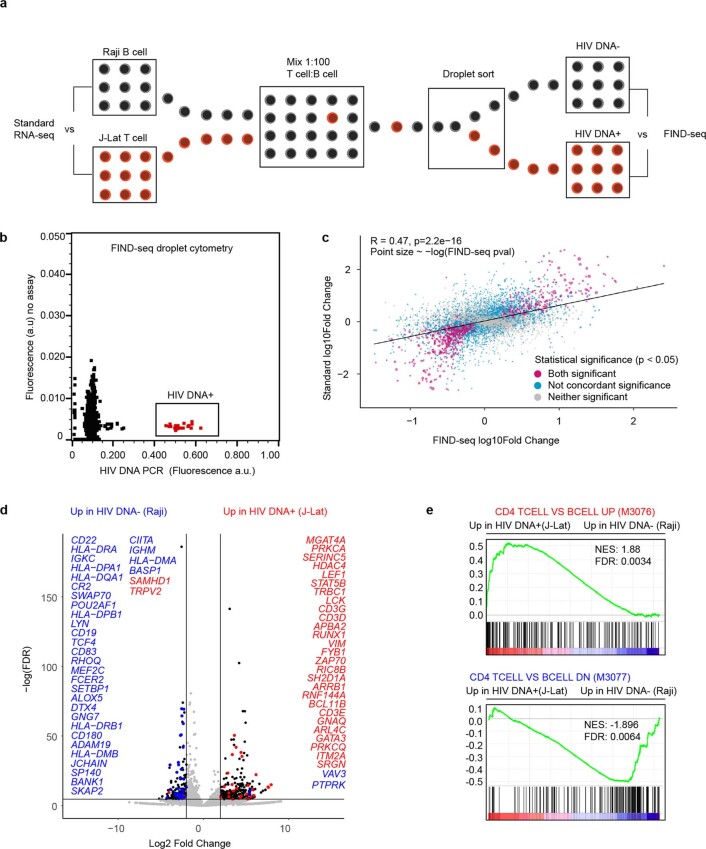
Validity of HIV DNA^+^ cell transcriptome sequencing after FIND-seq. (**a**) HIV DNA^+^ J-Lat T cells and HIV DNA^−^ Raji B cells were mixed at a 1:100 ratio and then subjected to FIND-seq and whole transcriptome sequencing. In parallel, extracted RNA samples from J-Lat cells and Raji cells were subjected to standard RNA-seq processing for comparison. (**b**) Droplet cytometry plot representing sorting of HIV DNA^+^ cells from the J-Lat:Raji mixture. (**c**) Correlation of gene expression differences between HIV DNA^+^ cells and HIV DNA^−^ cells (determined after FIND-seq) with gene expression differences between J-Lat cells and Raji cells (determined after standard RNA-seq). Points are coloured according to concordance of statistical significance in differential gene expression (DGE) results between FIND-seq and standard RNA-seq. Purple denotes genes significantly different (p < 0.05) in both FIND-seq and standard RNA-seq; grey denotes genes not significantly different in either FIND-seq or standard RNA-seq; blue denotes genes with discordant significance between standard and FIND-seq samples. Pearson’s R and p values were calculated in R v4.1.0. (**d**) Volcano plot of genes that were differentially expressed between HIV DNA^+^ and HIV DNA^−^ cells after FIND-seq, coloured by expected direction of change. Blue: differentially expressed genes found in gene set GSE10325 (M3077: CD4 T cell vs B cell down), red: differentially expressed genes found in GSE10325 (M3076: CD4 T cell vs B cell up). (**e**) Gene set enrichment pre-ranked analysis (GSEA) comparing transcriptomic differences between HIV DNA^+^ and HIV DNA^−^ cell samples sequenced from the J-Lat:Raji mixture after FIND-seq to previously reported differences between CD4 T cells and B cells (CD4 TCELL VS BCELL UP, M3076; CD4 TCELL VS BCELL DN, M3077).

We tested whether FIND-seq-sorted transcriptomes accurately represent the cells from which they are sorted by using mixtures of J-Lat T cells and Raji human B cells (Extended Data Fig. 2a). We cultured J-Lat and Raji cell lines separately and performed RNA sequencing (RNA-seq) analysis of each using standard protocols. At the same time, a 1:100 mixture of J-Lat and Raji cells was analysed using FIND-seq (Extended Data Fig. 2b). Gene expression differences between J-Lat and Raji cells after standard processing were highly correlated with differences between HIV-DNA^+^ and HIV-DNA^−^ cells after FIND-seq processing (*R* = 0.47, *P* = 2.2 × 10^−16^; Extended Data Fig. 2c). Furthermore, differential expression between J-Lat and Raji cells analysed using FIND-seq identified canonical T cell and B cell genes (Extended Data Fig. 2d) and agreed with published findings (Extended Data Fig. 2e). These results demonstrate that FIND-seq can be used to study the transcriptomic signatures of rare HIV-DNA^+^ cells.

## FIND-seq of HIV-DNA^+^ cells ex vivo

**Extended Data Fig. 3:**
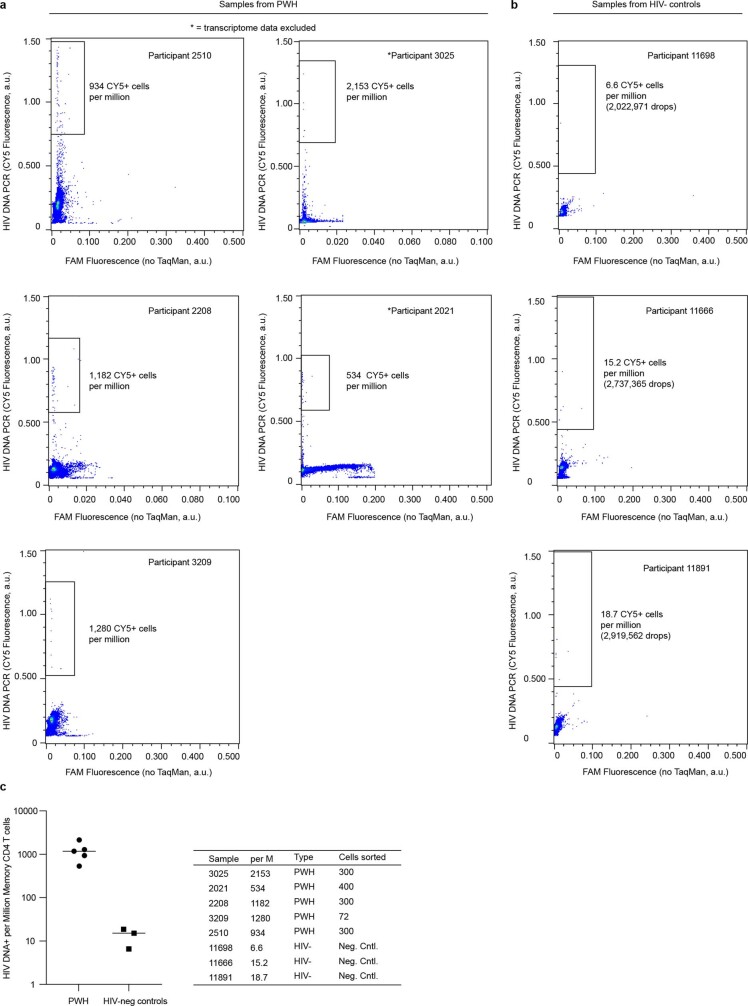
FIND-seq droplet cytometry of HIV DNA^+^ memory CD4 T cells *ex vivo*. (**a**) Droplet cytometry plots from the sorting of HIV DNA^+^ and HIV DNA^−^ memory CD4 T cells from five ART-treated people with HIV (PWH). Two PWH where transcriptome data were excluded from later analyses based on data quality are indicated with * (see “Participant sample data quality filtering” in Methods). (**b**) Negative control droplet cytometry plots of memory CD4 T cells from three HIV-uninfected participants. (**c**) Numbers of HIV DNA^+^ cells per million memory CD4 T cells, as measured by droplet cytometry during FIND-seq of samples from PWH and HIV-uninfected participants. *n* = 1 measurement for each of 5 PWH and each of 3 HIV-uninfected participants. Bars indicate median values. Total numbers of HIV DNA^+^ cells collected for each PWH are indicated in the table.

**Extended Data Fig. 4:**
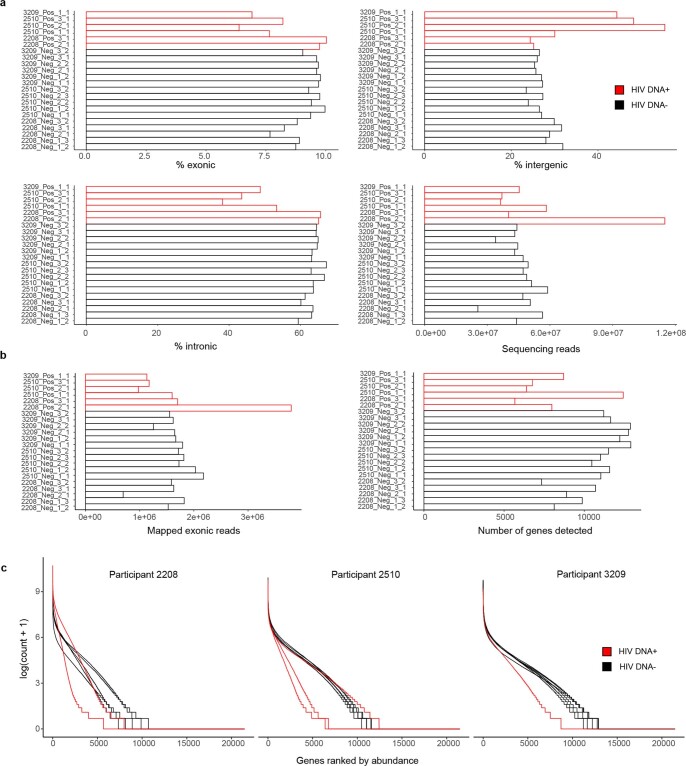
Transcriptome sequence composition and yield after FIND-seq from PWH. (**a**) Percentages of exonic, intergenic, and intronic reads, and total yields of sequencing reads, in curated samples. (**b**) Numbers of mapped exonic reads and genes detected in curated samples. (**c**) Rank abundance plots of genes in each sample, grouped by participant. Red bars and traces indicate HIV DNA^+^ samples, and black traces indicate HIV DNA^−^ samples.

To define gene expression patterns of HIV-DNA^+^ memory CD4 T cells under ART, we applied FIND-seq to magnetically purified memory CD4 T cell samples from five people with HIV receiving long-term ART that was initiated during chronic infection (Supplementary Table 1). Droplet cytometry data acquired during sorting demonstrated between 534 and 2,153 HIV-DNA^+^ cells per million (Extended Data Fig. 3a), consistent with previous studies using quantitative PCR analysis of extracted DNA^19,20^. False-positive frequencies of HIV-DNA^+^ memory CD4 T cells measured in three HIV-uninfected control participants ranged between 7 and 19 per million (Extended Data Fig. 3b). To maximize sorted transcriptome cDNA quantity and therefore reduce the need for extensive whole-transcriptome amplification (WTA) that could skew gene abundance in the sequencing libraries, we collected all droplets after HIV detection PCR in aliquots of 100 cell-equivalents. Sorting resulted in different numbers of aliquots collected across participants owing to the different frequencies of HIV-DNA^+^ cells (Extended Data Fig. 3c). After WTA and sequencing, we used a prospective curation process to select only those samples with a high library quality for further analysis (Methods). This resulted in a set of 22 curated samples from three people with HIV (Supplementary Table 2 and Extended Data Fig. 4).

## Host transcriptomes of HIV-DNA^+^ cells

**Fig. 2:**
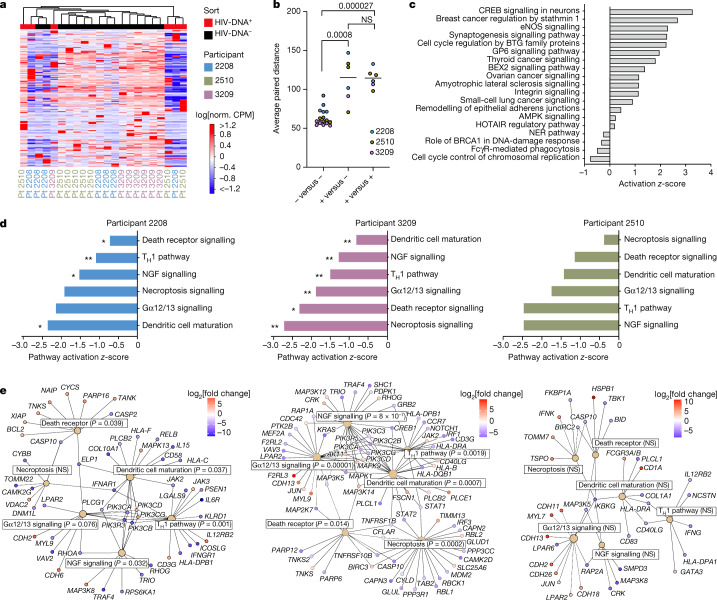
Host transcriptomic pathways in HIV-DNA^+^ memory CD4 T cells under ART. **a**, Unsupervised clustering analysis of HIV-DNA^+^ (red) and HIV-DNA^−^ (black) cell samples by expression of all protein-coding genes in the measured transcriptome. Norm., normalized; Pt, participant. **b**, The average Euclidean distances between sorted samples, defined by pairwise comparison of each HIV-DNA^−^ sample to all other HIV-DNA^−^ samples (negative versus negative); each HIV-DNA^+^ sample to all HIV-DNA^−^ samples (positive versus negative); and each HIV-DNA^+^ sample to all other HIV-DNA^+^ samples (positive versus positive). *n* = 16 biologically independent HIV-DNA^−^ and 6 biologically independent HIV-DNA^+^ samples sorted separately from three participants. Median values and *P* values calculated using Mann–Whitney *U*-tests are shown. **c**, Biological pathways enriched among DEGs (absolute fold change > 1.5 and FDR ≤ 0.05; Wald Test, Benjamini–Hochberg multiple-testing correction) between HIV-DNA^+^ and HIV-DNA^−^ cells in an analysis treating samples from distinct participants as biological replicates. *z*-scores for pathways with *P* < 0.05 (right-tailed Fisher’s exact test) are shown. **d**, Ingenuity pathways with a concordant direction of difference between HIV-DNA^+^ and HIV-DNA^−^ cells in all three participants analysed separately. Statistical analysis was performed using right-tailed Fisher’s exact tests; *P < 0.05, **P < 0.001. Numeric *P* values corresponding to the asterisks are shown in **e**. T_H_1, T helper 1 cells. **e**, Network plots showing DEGs identified separately in each participant that were part of shared Ingenuity pathways that were found to be inhibited in HIV-DNA^+^ cells across all three participants. Pathways are indicated by tan nodes labelled with the pathway name and *P* value; the node size is proportional to the number of DEGs identified within the pathway. Nodes indicating individual genes are coloured according to the relative expression between HIV-DNA^+^ and HIV-DNA^−^ cells (red, higher in HIV-DNA^+^ cells; blue, lower in HIV-DNA^+^ cells). Source data

Using the curated dataset (Supplementary Table 3), we first compared host gene expression between HIV-DNA^+^ and HIV-DNA^−^ memory CD4 T cells at the global level. Unsupervised clustering revealed partial segregation between HIV-DNA^+^ and HIV-DNA^−^ cell transcriptomes (Fig. 2a), and the use of Euclidean distance as a summary measure of transcriptomic relatedness demonstrated that distances between HIV-DNA^+^ and HIV-DNA^−^ cell samples were significantly greater than distances among HIV-DNA^−^ cell samples (*P *= 8.0 × 10^−4^; Fig. 2b). However, we also observed sample clustering by participant (Fig. 2a) as well as significantly greater Euclidean distances among HIV-DNA^+^ cell samples than among HIV-DNA^−^ cell samples (*P* = 2.7 × 10^−5^; Fig. 2b). We conclude that the whole-transcriptome clustering analysis suggested distinctive host gene expression by HIV-DNA^+^ memory CD4 T cells, but also indicated that transcriptomic differences among populations of HIV-DNA^+^ cells and across study participants are substantial sources of variation in the dataset.

## Host gene differential expression

**Extended Data Fig. 5:**
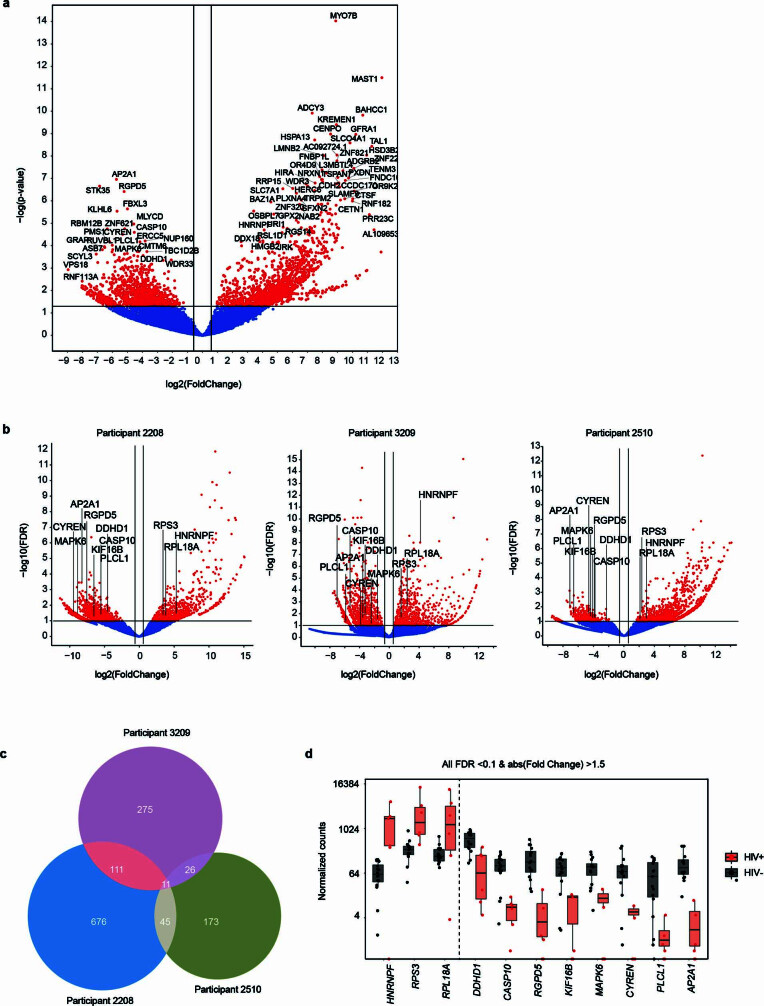
Host gene expression by HIV DNA^+^ and HIV DNA^−^ memory CD4 T cells under ART. (**a**) Volcano plot of DGE between HIV DNA^+^ and HIV DNA^−^ cells from DGE analysis that considered samples from participants as biological replicates. Genes showing Fold Change >1.5 and FDR ≤0.05 between HIV DNA^+^ and HIV DNA^−^ cells are highlighted in red. (**b**) DGE between HIV DNA^+^ and HIV DNA^−^ cells, analysed separately in each participant. Genes with absolute fold change >1.5 and p ≤0.1 are highlighted in red. Labels indicate DEGs that were common to all three participants. (**c**) Overlap of DEGs (absolute fold change ≥1.5, p ≤0.1) between HIV DNA^+^ and HIV DNA^−^ after separate analysis of each participant. (**d**) RNA expression of DEGs that were common to all participants. Each plotted point indicates the expression level of the given gene in a single sorted sample (*n* = 16 biologically independent HIV DNA^−^ and 6 biologically independent HIV DNA^+^ samples). Box plots indicate the median with the lower and upper hinges corresponding to the 25th and 75th percentiles and whiskers corresponding to 1.5 x the interquartile range.

To identify individual genes and transcriptomic pathways that were characteristic of HIV-DNA^+^ memory CD4 T cells, we performed differential gene expression (DGE) analysis using two distinct approaches (Supplementary Table 4). Using a combined approach that analysed participants as biological replicates, we identified 2,776 differentially expressed genes (DEGs; absolute fold change > 1.5, FDR ≤ 0.05) (Extended Data Fig. 5a). Pathway enrichment analysis on the basis of these DEGs yielded several cancer- and cell-cycle-related pathways (Fig. 2c), suggesting differences between HIV-DNA^+^ and HIV-DNA^−^ memory CD4 T cells related to cell proliferation and survival. Notably, a comparison of DEG lists defined for each of the participants separately revealed only 11 DEGs common to all three participants (Extended Data Fig. 5b–d). However, pathway enrichment analysis using participant-specific DEG lists (absolute fold change ≥ 2, *P* ≤ 0.01) identified six pathways that shared concordant direction across participants (Fig. 2d and Supplementary Table 5). All six concordant pathways showed *z*-activation scores of <0, indicating pathway inhibition in HIV-DNA^+^ cells relative to HIV-DNA^−^ cells. Notably, these inhibited pathways in HIV-DNA^+^ cells included death receptor signalling, necroptosis signalling and the anti-proliferative Gα12/13 signalling pathway^33^. Inferences of pathway inhibition arose from both decreased expression of pathway activators and increased expression of pathway inhibitors in HIV-DNA^+^ cells and depended on differential expression of distinct pathway genes in different participants (Fig. 2e). We conclude that although many individual DEGs distinguishing HIV-DNA^+^ cells from HIV-DNA^−^ cells differed between the participants, higher-order analysis revealed that inhibition of cell death and anti-proliferative signalling are shared attributes of HIV-DNA^+^ memory CD4 T cells under ART.

## Analysis of co-expressed gene signatures

**Fig. 3:**
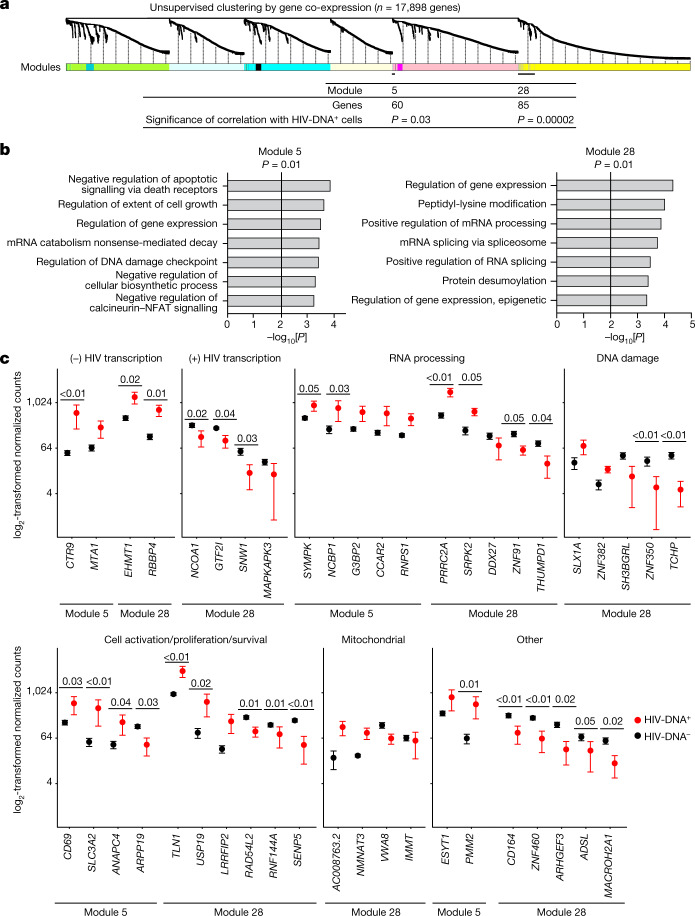
Co-expressed gene signatures in HIV-DNA^+^ memory CD4 T cells under ART. **a**, The 17,898 genes detected in ≥2 samples from the curated dataset were processed for WGCNA as described in the Methods. A total of 28 resulting modules of genes defined by distinct co-expression patterns across the samples are indicated as coloured segments, with the relatedness among genes indicated by the dendrogram. The two modules that were significantly correlated with the HIV DNA status of the samples are indicated at bottom (module 5, 60 genes, *R* = 0.46, *P* = 0.03; module 28, 85 genes, *R* = 0.78, *P* = 2 × 10^−5^; weighted Pearson correlation). **b**, Gene ontology (GO) analysis of module 5 and module 28 gene lists. All significant terms (adjusted *P* ≤ 0.05, Fisher’s exact test, Benjamini–Hochberg multiple-testing correction) are shown, except for redundant terms that are shown in Supplementary Table 6, but were omitted here for clarity. **c**, Genes from modules 5 and 28 with normalized expression levels that differed by an average of at least twofold between HIV-DNA^+^ and HIV-DNA^−^ cells and had a concordant direction of difference in all of the participants. Genes are grouped in individual plots according to putative biological function. *P* values calculated using Wald tests are shown for genes with *P* < 0.05 in the differential expression analysis between HIV-DNA^+^ and HIV-DNA^−^ cells. *n* = 16 biologically independent HIV-DNA^−^ and 6 biologically independent HIV-DNA^+^ samples sorted separately from 3 participants. Data are mean ± s.e.m.

We anticipated that pooled sequencing from diverse HIV-DNA^+^ memory CD4 T cells under ART could dilute signals from infected cell subpopulations, thereby limiting the detection of informative features of HIV-infected cells in conventional DGE analysis. To identify transcriptomic signatures of HIV-DNA^+^ cells as groups of genes, we used weighted gene co-expression network analysis (WGCNA) to define gene modules on the basis of correlation patterns across samples (Supplementary Table 6). Within the curated set of 22 samples that together expressed 17,898 different genes, this process produced 28 distinct gene modules of varying sizes (Fig. 3a). Correlating module gene expression patterns with cell infection status (that is, HIV-DNA^+^ versus HIV-DNA^−^) identified significant correlations for module 5 (60 genes, *R* = 0.46, *P* = 0.03) and module 28 (85 genes, *R* = 0.78, *P* = 2 × 10^−5^) (Fig. 3a). Thus, unsupervised clustering using WGCNA revealed two groups of genes that account for only 0.81% of the measured transcriptome that distinguished HIV-DNA^+^ from HIV-DNA^−^ memory CD4 T cells in ART-treated people with HIV.

To characterize the differences between HIV-DNA^+^ and HIV-DNA^−^ memory CD4 T cells reflected by these modules, we analysed the module gene lists using Gene Ontology (GO). In both modules, we found statistically significant enrichment (adjusted *P* ≤ 0.05) for genes related to the regulation of gene expression at the transcriptional and post-transcriptional levels (Fig. 3b). Module 28 was enriched for GO terms related to mRNA splicing and processing. Module 5 was enriched for genes involved in mRNA degradation by nonsense-mediated decay, which has been linked to negative post-transcriptional regulation of HIV gene expression in vitro^34^. Moreover, module 5 was enriched for terms related to cell survival, activation and proliferation, including regulation of death receptor signalling, regulation of calcineurin–NFAT signalling and DNA-damage checkpoint regulation. We conclude that GO analysis of WGCNA module genes identified transcriptional and post-transcriptional gene regulation as well as several cell state regulatory processes as distinguishing attributes of HIV-DNA^+^ memory CD4 T cells under ART.

Furthermore, we examined the transcriptomic differences between HIV-DNA^+^ and HIV-DNA^−^ memory CD4 T cells by inspecting a filtered list of the 44 genes in WGCNA modules 5 and 28 that showed at least twofold average difference between HIV-DNA^+^ and HIV-DNA^−^ cell populations and a concordant direction between populations across the participants (Fig. 3c, Extended Data Table 1 and Supplementary Table 6). We noted that 8 out of 44 genes were previously implicated in the regulation of HIV transcription. Four genes were linked to negative regulation of HIV transcription through histone modification (*EHMT1*^35^, *RBBP4*^36^ and *MTA1*^37^) or promoter-proximal pausing of RNA polymerase II (*CTR9*^38^), and were higher in HIV-DNA^+^ cells. The remaining four genes were linked to positive regulation of HIV transcriptional initiation (*GTF2I*^39^ and *MAPKAPK3*^40^) or elongation (*NCOA1*^41^ and *SNW1*^42^), and were lower in HIV-DNA^+^ cells. We conclude that host gene expression signatures of HIV-DNA^+^ memory CD4 T cells under ART were relatively non-permissive for HIV transcription.

We next examined the remaining 36 genes from the filtered module 5 and 28 gene lists. Ten of these genes encoded RNA-processing factors. In module 5, these included higher levels in HIV-DNA^+^ cells of antiviral defence factor *NCBP1*^43^ and post-splicing complex component *RNPS1*^44^, both of which have been linked to nonsense-mediated decay. Module 5 also included higher levels in HIV-DNA^+^ cells of *G3BP2*, a stress granule factor in a gene family that has been implicated in cytoplasmic sequestration and translational inhibition of HIV mRNAs^45^. mRNA-processing factors in module 28 included higher levels in HIV-DNA^+^ cells of *PRRC2A*—a reader of *N*^6^-methyladenosine RNA modifications that can be induced by HIV infection in vitro^46^—and the splicing regulator SRPK. Among the additional 26 genes, we noted that module 28 included *USP19* and *LRRFIP2*, which can inhibit apoptosis^47^ or pyroptosis^48^ and were higher in HIV-DNA^+^ cells, and *TLN1*^49^, which is required for antigen-driven T cell proliferation mediated through immunological synapses^49^ and was also higher in HIV-DNA^+^ cells. Finally, we noted multiple module 28 genes involved in the DNA-damage response and mitochondrial function. We conclude that the transcriptomic signatures of HIV-DNA^+^ memory CD4 T cells under ART suggest that these cells have the capacity for post-transcriptional HIV silencing, and are also consistent with DGE-based indications of increased cell survival and proliferation.

## Enrichment of signatures in cell subsets

**Extended Data Fig. 6:**
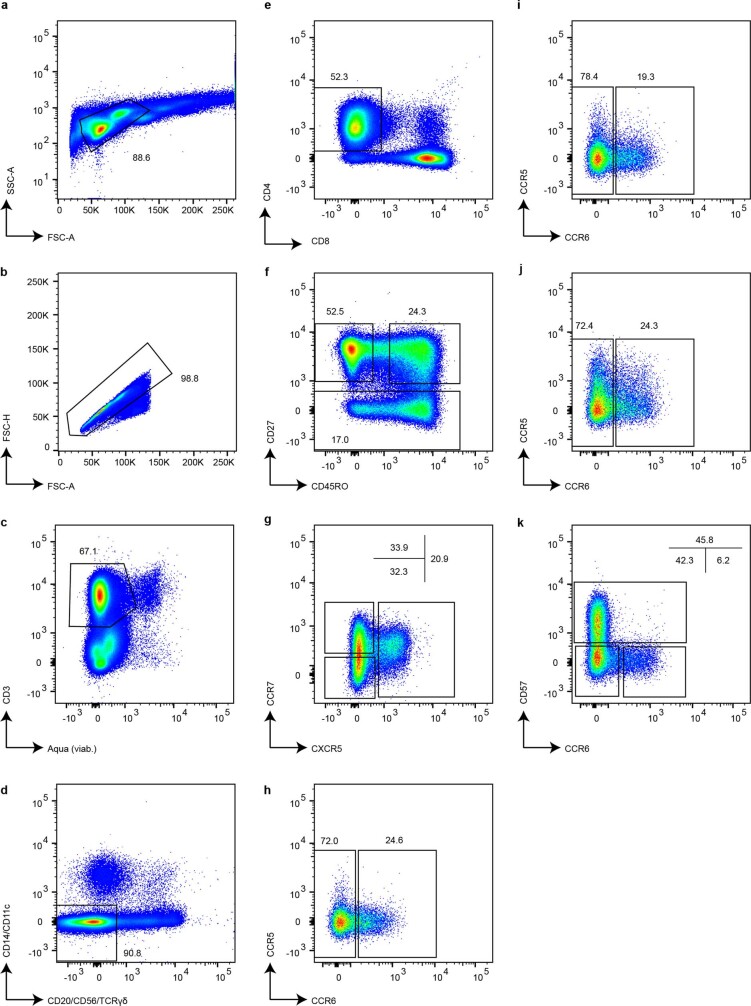
Fluorescence-activated cell sorting of circulating CD4 T cell subsets in ART-treated PWH. Leukocytes (**a**) not part of multi-cell conjugates (**b**) that were viable and stained with the T cell marker CD3 (**c**) but not lineage markers CD20, CD56, TCR-γδ, CD14, or CD11c (**d**) and were CD4^+^ and CD8^−^ (**e**) were identified by CD27 and CD45RO staining as phenotypically naïve (**f**, top-left gate, CD27^+^CD45RO^−^ population) or memory (**f**, top-right and bottom gates) CD4 T cells. CD27^+^ memory CD4 T cells (**f**, top-right gate) were further separated into three populations by CXCR5 and CCR7 expression (**g**). Each of these three populations was then collected in two subsets defined by CCR6 expression (**h**–**j**). CD27^−^ memory CD4 T cells (**f**, bottom gate) were collected in three subsets defined by CCR6 and CD57 expression (**k**). The sorting strategy yielded purified naïve and 9 subsets of memory CD4 T cells. The marker expression patterns of the sorted memory CD4 T cell subsets are shown in Extended Data Table 2. Percentages of all events on each plot falling within the indicated gates are indicated. Results are shown for participant ID 2013.

To investigate the origins of HIV-DNA^+^ memory CD4 T cell transcriptomic signatures identified by co-expression network analysis, we compared these signatures with the transcriptomes of defined CD4 T cell subsets. We isolated circulating naive and memory CD4 T cell subsets from nine ART-treated people with HIV (Supplementary Table 1) using fluorescence-activated cell sorting (FACS) (Extended Data Fig. 6), defined subset gene expression using RNA-seq and finally used gene set enrichment analysis (GSEA) to compare gene expression signatures in the sorted memory subsets (defined by expression relative to the naive subset) against co-expression network analysis signatures of HIV-DNA^+^ cells (Extended Data Table 2). This revealed significant enrichment of the module 5 signature in memory CD4 T cells of the CD27^+^CCR7^+^CD45RO^+^CXCR5^+^CCR6^−^ peripheral T follicular helper (T_FH_) phenotype^50^. No significant enrichment was observed for the module 5 signature in any other subset, or for the module 28 signature in any of the subsets. We conclude that, taken together, the transcriptomic signatures of HIV-DNA^+^ memory CD4 T cells under ART did not map to defined CD4 T cell subsets, although the module 5 signature showed partial similarity to the signature of CCR6^−^ peripheral T_FH_ cells in ART-treated people with HIV.

## HIV RNA expression analysis

**Fig. 4:**
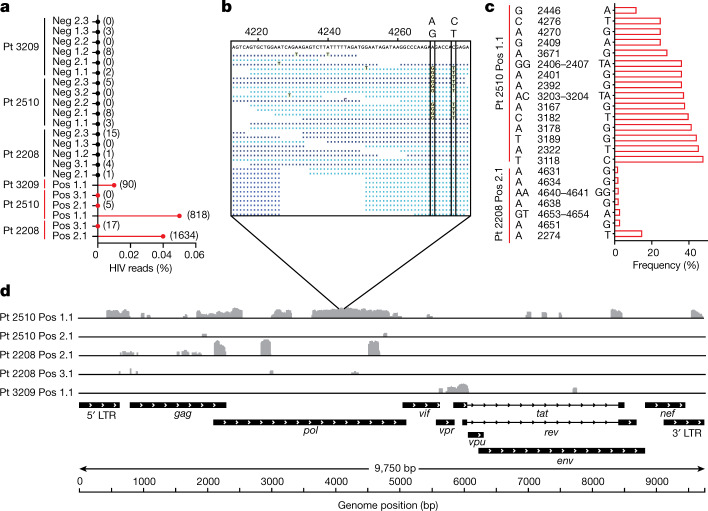
HIV RNA sequences in memory CD4 T cells under ART. **a**, The percentages of all exonic reads mapping to a clade B HIV reference genome for all samples. Absolute read counts are shown in parentheses. HIV-DNA^−^ samples are shown in black, and HIV-DNA^+^ samples are shown in red. **b**, Expanded view of coverage for sample 2510 Pos 1.1, with the vertical boxes indicating two linked variant positions within the given region. Reads shown in light blue and dark blue are those mapped in the forward and reverse orientations. Nucleotide bases that match the sample consensus at the top are shown as dots. **c**, Graphical representation of HIV reference genome base positions at which variation was detected among sequence reads, as described in the Methods. Samples with no detectable variation are not shown. **d**, The coverage of mapped reads across the HIV reference genome for HIV-DNA^+^ cells. Individual samples are labelled with the participant ID number, followed by the HIV DNA status (HIV-DNA^+^ (pos) and HIV-DNA^−^ (neg)), and then an identifier denoting the replicate number. Sample 2510 Pos 3.1 is omitted from this panel owing to a lack of HIV reads in that sample.

Finally, we used the curated set of 22 samples to analyse HIV transcriptional patterns in HIV-DNA^+^ memory CD4 T cells under ART by aligning transcriptome sequence reads to a reference HIV genome (Fig. 4). We found that some HIV-DNA^+^ cell samples showed hundreds of HIV reads (Fig. 4a), including one sample from participant 2510 with two distinct virus sequences (Fig. 4b,c) that suggested processive HIV transcripts from at least two cells in the sorted aliquot of 100 cells. Nevertheless, HIV read percentages for all HIV-DNA^+^ cell samples were <0.05% (Fig. 4a), which is 100-fold lower than previously reported for HIV-expressing cells sequenced after in vitro stimulation^51^. These findings are consistent with latent infection and/or HIV sequence defects that limit virus transcription in HIV-DNA^+^ cells. HIV genome coverage patterns of mapped reads were notable for isolated peaks interspersed with areas of no coverage (Fig. 4d), suggesting atypical transcription start sites^52^, transcripts from proviruses with deletion mutations and/or chance sampling variations. Spliced transcripts were not detected even by manually inspecting and mapping individual mates of read pairs using BLAST. The use of assembly-based tools to produce contigs from reads that did not initially map to the human reference yielded no HIV contigs from 5/6 HIV-DNA^+^ cell samples and did not substantially increase mapped HIV read counts in the remaining sample (not shown). We conclude that polyadenylated RNA-seq in HIV-DNA^+^ memory CD4 T cells from ART-treated people with HIV did not reveal either full-length genomic HIV transcripts or spliced HIV messages encoding accessory proteins.

## Discussion

The absence of evidence for HIV reservoir size reduction in ‘shock and kill’ clinical trials has bred uncertainty about the role of therapeutic HIV latency reversal and the use of the latent reservoir concept. Meanwhile, attempts to understand the mechanisms of HIV persistence under ART by identifying distinctive attributes of HIV-infected CD4 T cells have faced major technical obstacles. Using microfluidic technology developed to study HIV-DNA^+^ memory CD4 T cells under ART in their natural state, we identified host gene expression signatures in these rare cells that were intrinsically non-permissive for the transcription of the virus. This supports the concept that these cells are a latent reservoir and links HIV transcriptional quiescence in vivo to host gene expression patterns that are specific to infected cells. Furthermore, host transcriptomic signatures of HIV-DNA^+^ memory CD4 T cells under ART indicated that the persistence of these cells may involve additional mechanisms beyond HIV transcriptional silencing, including post-transcriptional HIV silencing, resistance to cell death and resistance to anti-proliferative signalling. These findings are consistent with incomplete latency reversal by early LRAs^53–58^ and the persistence of infected cells observed even after cell stimulation both in vitro^59^ and in vivo^12–16^. Overall, our results in this study therefore reveal a host cell transcriptomic signature of which further elucidation may lead to the development of new HIV cure strategies.

The origins of the gene expression patterns that we identified in this study will require further investigation. In part, these patterns may arise progressively under ART through the selective elimination of cells that do not express them. Selection for an HIV-silencing signature may occur among cells that are competent to express toxic virus gene products in vivo, while selection for cell survival and proliferation could apply to the entire HIV-DNA^+^ cell pool. Importantly, this selection model implies that there are pre-existing differences among CD4 T cells in the expression of HIV silencing, cell survival and cell proliferation signatures that did not trace in their entirety to a single memory CD4 T cell subset. These signatures may therefore reflect mixed contributions from multiple subsets, each with modest enrichment for the virus, perhaps exemplified by our partial mapping of one co-expressed module signature to peripheral T_FH_ cells. At the same time, it is also possible that some gene expression patterns of HIV-infected memory CD4 T cells are a consequence of HIV infection in these cells. Cellular transcriptomic reprogramming could represent a host response to HIV integration or other life cycle steps, as suggested by co-expressed module signature genes encoding virus-induced and DNA-damage response factors. Alternatively, although we detected little evidence of polyadenylated HIV RNA expression in HIV-DNA^+^ cells, it remains possible that components of infecting HIV virions or HIV expression products of which transcripts went undetected in our sequencing—due to transient expression or method sensitivity—might actively reprogram host cell gene expression. Future studies elucidating such mechanisms may yield new targets for HIV cure strategies.

Our findings in this study have several limitations. First, owing to technical challenges, we sorted and sequenced pools of HIV-DNA^+^ cell transcriptomes without distinguishing between intact and defective HIV genomes^31^. As a result, technical advances in FIND-seq and/or new technologies will be required to define how the transcriptomic signatures identified here are distributed among individual cells. Analysis of HIV-DNA^+^ cells at the single-cell level will avoid dilution of signatures from reservoir subpopulations, thereby refining and extending the findings from this study. Single-cell transcriptomic analyses that distinguish between intact and defective HIV may also clarify whether HIV silencing signatures arise strictly by selection within translation-competent reservoirs, or whether these signatures can arise even when the infecting virus genome has acquired lethal mutations during reverse transcription. Second, although many of the transcriptomic signature genes identified here have well-defined roles in regulating HIV gene expression, cell survival or cell proliferation, the roles of other genes in HIV persistence will require further study. Those signature genes that have RNA-processing functions but have not previously been linked with HIV replication will be of particular interest, as some of these could contribute to post-transcriptional regulation of HIV gene expression while others might serve only as markers of infected cells. Third, our findings address neither the durability of transcriptomic signature expression within each infected cell nor the distribution of cells expressing signature genes across diverse tissue compartments, raising important questions about reservoir cell dynamics that impact the development of HIV cure strategies. Fourth, as our study included a small number of participants, it is possible that larger FIND-seq studies performed in diverse participant populations and incorporating technical improvements to increase the recovery of high-quality data will reveal signatures that were not detected here. Finally, it is important to acknowledge that the barriers to HIV cure under ART may include virus reservoirs outside the memory CD4 T cell pool^60–62^.

Notwithstanding these limitations, our findings highlight two parallel but complementary paths in translational and basic research towards an HIV cure. The first is an increased emphasis on in vivo studies targeting the full range of mechanisms that both maintain HIV quiescence and prevent the death of HIV-infected cells. The approaches taken may include synergistic combinations of LRAs targeting diverse HIV transcriptional and translational blocks, paired with therapies that potentiate physiological CD4 T cell death. However, as the complexity of therapeutic combinations increases, their potential for significant toxicity may become a growing concern. Thus, the second path forward is an ongoing effort to define gene expression patterns within HIV-infected cellular reservoirs and to understand their mechanistic basis. The intent is for these approaches to reveal how HIV silencing, cell survival and cell proliferation programs come to be expressed among the diverse memory CD4 T cells present in vivo, therefore generating additional insights that may be translated to effective and safe HIV-cure-directed therapies.

## Methods

### Study participants

Recruitment of study participants with HIV was performed in compliance with relevant ethical regulations under the IRB-approved SCOPE protocol (NCT00187512) at San Francisco General Hospital. Participants were enrolled from the SCOPE cohort on the basis of sample availability at the time of study, without use of sample size calculations, blinding or randomization. Demographic and clinical laboratory data were collected at San Francisco General Hospital and are reported in Supplementary Table 1. All of the participants provided informed consent before study. Prescreening of participant samples to ensure adequate numbers of HIV-DNA^+^ memory CD4 T cells for FIND-seq analysis was performed in parallel sample aliquots using fluorescence-assisted clonal amplification^63^.

### Cell lines

Jurkat human T cells (TIB-152, ATCC), HIV-DNA^+^ J-Lat full-length human T cells (clone 6.3, ARP-9846)^64^ and Raji human B cells (CCL-86, ATCC) were cultured in Gibco RPMI Medium 1640 (Thermo Fisher Scientific, 11875093) with penicillin and streptomycin (Thermo Fisher Scientific, 15140122) and 10% fetal bovine serum (FBS). Mouse fibroblasts (NIH/3T3, CRL-1658, ATCC) were cultured in Dulbecco’s modified Eagle’s medium (DMEM) with penicillin and streptomycin (Thermo Fisher Scientific, 15140122) and 10% FBS. Before use, 3T3 cells were dissociated using 0.25% trypsin-EDTA (Thermo Fisher Scientific, 25200-072) and neutralized in DMEM with 10% FBS. Cell lines were used without authentication or mycoplasma contamination testing.

### Fabrication of microfluidic devices

Standard photolithography techniques were used to fabricate microfluidic devices at the Harvard Medical School Microfluidics Facility. Silicon wafers were spin-coated with SU-8 2025/2050 photoresist (Kayaku Advanced Materials) and ultraviolet-patterned using a mask aligner. After developing, the wafers were baked overnight and used as master moulds for soft-lithography. In brief, the PDMS prepolymer and curing agent were mixed by hand at a ratio of 10 to 1 (Momentive, RTV615), degassed for at least 1 h, poured onto the mould and degassed until no bubbles remained. PDMS was baked overnight at 65 °C before holes were punched using a 0.75 mm biopsy punch and bonded to a glass slide (75 × 50 × 1.0 mm, Thermo Fisher Scientific, 12–550C) with a plasma bonder (Technics Plasma Etcher 500-II). Bonded devices were made hydrophobic with Aquapel with a 30 s contact time, flushed with HFE-7500, purged with air and baked for at least 1 h before use.

### Cell line validation studies

Cells were washed twice with Hanks’ balanced salt solution (HBSS, no calcium, no magnesium, Thermo Fisher Scientific, 14170112) and then counted, mixed (mouse:human 1:1; J-Lat:Raji 1:100), and resuspended in HBSS containing 18% OptiPrep Density Gradient Medium (Sigma-Aldrich) for FIND-seq. For standard RNA-seq studies performed in parallel, aliquots of 5 × 10^4^ cells were lysed in RNAzol RT (Molecular Research Center) and stored at −80 °C until subsequent total RNA extraction according to the manufacturer’s instructions. Whole-transcriptome cDNA was then generated from total RNA by reverse transcription using 6 mM MgCl_2_, 1 M betaine, 7.5% PEG-8000, 1 mM dNTP, 2 U µl^−1^ Maxima H-minus reverse transcriptase (Thermo Fisher Scientific, EP0753), 0.5 U µl^−1^ RNase inhibitor (Lucigen, NxGen) and 2 µM SMART TSO (AAGCAGTGGTATCAACGCAGAGTGAATrGrGrG). This cDNA was purified using AMPure XP beads (Beckman Coulter), and was then processed for WTA by PCR, with library preparation as previously described^65^. FIND-seq sample processing and library preparation were performed as described below. The correlation between the DGE results from standard RNA-seq and FIND-seq was analysed using stat_cor (method = “pearson”) in R (v.4.1.0). The results from the J-Lat:Raji mixing study were compared with published transcriptomic signatures of CD4 T cells and B cells^66^ using GSEA.

### PBMC processing for FIND-seq

Approximately 20–30 million cryopreserved peripheral blood mononuclear cells (PBMCs) from each study participant were used for FIND-seq. Cryopreserved PBMC suspensions were thawed in a 37 °C water bath, washed in prewarmed RPMI with 10% FBS, and sedimented by centrifugation at 300 rpm (Sorvall Legend XT). Untouched memory CD4 T cells were then isolated by magnetic-column-based negative selection (Miltenyi, 130-091-893). Cells were counted manually with a haemocytometer using Trypan blue, and aliquots of 5 × 10^4^ cells were lysed and stored in RNAzol RT.

### FIND-seq

FIND-seq was performed as described previously^30^. In brief, four syringes were prepared for microfluidic cell encapsulation: lysis buffer, agarose, cells and oil. The lysis buffer consisted of 20 mM Tris-HCl pH 7.5, 1,000 mM LiCl, 1% LiDS, 10 mM EDTA, 10 mM DTT and 0.4 µg µl^−1^ proteinase K. Conjugated agarose-dT was heated to 95 °C for 1 h before use and was kept heated throughout the run using a custom syringe heater. A 10 ml syringe was loaded with oil (Bio-Rad, 186–3005) for droplet generation. All of the syringes were connected to the microfluidic device using PE/2 tubing (Scientific Commodities, BB31695-PE/2). To make droplets, pumps were run at 600 μl h^−1^ (cell mixture), 1,200 μl h^−1^ (agarose), 600 μl h^−1^ (lysis buffer), and 5,000 μl h^−1^ (oil) using a bubble-triggered drop generator^67^. Air was controlled to break the jet and generate 53–55 µm droplets. After lysis at 55 °C for 2 h, droplets were cooled at 4 °C overnight to allow agarose gelation. Solid agarose microspheres (beads) were removed from the oil using a drop-breaking procedure. All of the steps were performed at 4 °C to prevent dissociation of mRNA from the poly(T) oligonucleotides. The beads were removed from the oil and washed five times. For each wash, the beads were incubated in wash buffer for 5 min on ice, centrifuged at 4,700 rpm for 10 min and aspirated before the next wash. Beads were first washed in wash buffer 1 containing 20 mM Tris-HCl pH 7.5, 500 mM LiCl, 0.1% LiDS and 0.1 mM EDTA. Next, the beads were washed twice with wash buffer 2 containing 20 mM Tris-HCl pH 7.5 and 500 mM NaCl. Finally, the beads were washed twice in 5× reverse transcription buffer containing 250 mM Tris-HCl pH 8.3, 375 mM KCl, 15 mM MgCl_2_ and 50 mM DTT and filtered with a 100 µm cell strainer. The beads were resuspended in reverse transcription master mix to a final concentration of 6 mM MgCl_2_, 1 M betaine, 7.5% PEG-8000, 1 mM dNTP, 2 U µl^−1^ Maxima H-minus reverse transcriptase (Thermo Fisher Scientific, EP0753), 0.5 U µl^−1^ RNase inhibitor (Lucigen, NxGen) and 2 µM SMART TSO (AAGCAGTGGTATCAACGCAGAGTGAATrGrGrG). Reverse transcription was completed at 25 °C for 30 min, followed by 90 min at 42 °C. The tubes were mixed continuously with an inverter during all incubations. After reverse transcription, the beads were washed five times with 0.1% Pluronic in RNase/DNase-free water.

After reverse transcription, the cell occupancy of agarose beads was quantified by microscopy and successful reverse transcription was checked using WTA before continuing with bead reinjection and sorting. Agarose beads containing cellular genomes and transcriptomes were reinjected into droplets to perform single-cell HIV detection PCR. Beads were mixed with PCR reagents to achieve a final concentration of 1× TaqPath Mastermix (Thermo Fisher Scientific, A30866), PEG-6000 (0.5% (w/v)), Tween-20 (0.5% (w/v)), F-127 Pluronic (0.5% (w/v)), BSA (0.1 mg ml^−1^), HIV *gag* forward primer (CACTGTGTTTAGCATGGTGTTT, 900 nM), HIV *gag* reverse primer (TCAGCCCAGAAGTAATACCCATGT, 900 nM) and HIV *gag* hydrolysis probe (CY5-ATTATCAGAAGGAGCCACCCCACAAGA-3′ Iowa Black RQ, 250 nM)^68^. To generate the final 1× reaction mixture concentration, beads were soaked in 2× PCR master mix on a shaker for 30 min in the dark. Next, the beads were centrifuged and loaded into a 3 ml syringe. The remaining 1× PCR master mix (supernatant) was loaded into a separate 3 ml syringe. Finally, the beads and 1× PCR master mix were reinjected in the microfluidic device to encapsulate the beads into 70 µm droplets^69^. Agarose beads were re-encapsulated in droplets with about 70% loading, which is not accounted for in the detection efficiency calculation. Droplets were collected in 40 µl aliquots in PCR strips and thermocycled as follows: 88 °C for 10 min; then 55 cycles of 88 °C for 30 s and 60 °C for 1 min. After thermocycling, droplets were transferred into a 3 ml syringe for microfluidic sorting.

HIV-DNA^+^ and HIV-DNA^−^ droplets were sorted on the basis of the HIV PCR signal using a concentric sorter as previously described^32^. For HIV-DNA^−^-sorted samples, we sorted 100 cell equivalents based on the number of genomes per hydrogel bead determined previously, collecting a mixture of HIV-DNA^−^ cell droplets and cell-free droplets. For HIV-DNA^+^-sorted samples, we sorted aliquots of 100 droplets. The sorter was run with the following flow rates: 180 μl h^−1^ cell droplets, 6,000 μl h^−1^ bias oil (HFE-7500), 250 μl h^−1^ spacer oil (HFE-7500) and 3,500 μl h^−1^ extra spacer oil (HFE-7500). To sort, the 2 M NaCl on-chip electrode was polarized using a high-voltage amplifier at 1,200 V, 4,000 Hz for 15 cycles with 120 μs delay. We sorted into 1.5 ml Eppendorf tubes, removed all but 20 µl of the oil, added 50 µl of distilled nuclease-free water and centrifuged the sample at 20,000*g* for 5 min, and then stored the samples at −80 °C.

Before performing WTA on sorted HIV-DNA^+^ droplets in each participant, we determined the WTA cycle number that was required to amplify transcriptome cDNA from 100 cells in that participant. Accordingly, we first performed WTA on HIV-DNA^−^-sorted sample aliquots. Sorted HIV-DNA^−^ sample aliquots (frozen at −80 °C) were heated to 60 °C on a heat block for 10 min, mixed carefully by pipet and centrifuged at 20,000*g* for 5 min. The aqueous layer was then transferred to PCR strips and a WTA PCR reaction was performed using the 1× KAPA HiFi Master mix (Roche, KK2601) and 0.4 μM Smart-seq2 primer (AAGCAGTGGTATCAACGCAGAGT). Sorted material was thermocycled as follows: 95 °C for 3 min; then 18–22 cycles of 98 °C for 15s, 67 °C for 20s and 68 °C for 4 min; then 72 °C for 5 min, with a 4 °C terminal hold. The WTA was performed at three different cycle numbers—18, 20, and 22 cycles. All reactions were subsequently purified using a 1.2:1 ratio of AMPure XP beads (Beckman Coulter), with the final elution performed in 20 µl of nuclease-free water. After WTA, the DNA yield was quantified using the Qubit 4 Fluorometer and DNA size distribution was assayed using a Bioanalyzer 2100 with High Sensitivity DNA chip. On the basis of these results, the HIV-DNA^+^-sorted samples were processed as above using the minimal cycle number required to achieve a concentration of greater than 2 ng µl^−1^ in 20 µl of elution volume.

### Sequencing and read preprocessing

Libraries were prepared from transcriptome material sorted by FIND-seq using the Nextera XT Library Preparation Kit with v2 indexes. Individual sample libraries were combined at equimolar amounts to produce a single library pool. The library was quantified using the KAPA SYBR FAST Universal qPCR Kit. The library concentration and fragment size distribution were confirmed using the Agilent Bioanalyzer 2100 with High Sensitivity DNA chip. The library was diluted and denatured in accordance with the Illumina MiSeq System Denature and Dilute Libraries Guide (document 15039740). Cell line libraries were sequenced on the Illumina MiSeq system in 2 × 75 bp runs, and the selected libraries were subsequently sequenced again on the Illumina HiSeq 4000 system in a 2 × 75 bp run, operated using the Illumina HiSeq Control Software (HCS) v.3.4.0. For samples from people with HIV, libraries were first pooled and run on the Illumina MiSeq system in a 2 × 75 bp run, then rebalanced and run on the Illumina HiSeq 4000 system in a 2 × 75 bp run. Raw sequencing data were converted to fastq format using the bcl2fastq2 script (v.2.20) from Illumina and the reads were demultiplexed using sample-specific indexes. The resulting fastq files were trimmed for quality, ambiguity and presence of read-through adapters using the ‘Trim reads’ tool with the default settings in CLC Genomics Workbench (GWB) v.21.0.3. The quality of the raw and trimmed reads was assessed using QC tools in GWB.

### Participant sample data quality filtering

Owing to the abundance of HIV-DNA^−^ cells in samples from ART-treated people with HIV, HIV-DNA^−^ cells were sorted in multiple replicates. Sequencing data were generated from 53 HIV-DNA^+^ and HIV-DNA^−^ cell samples sorted by FIND-seq from 5 people with HIV. A prospective curation approach was used to exclude low-quality samples from downstream transcriptomic analysis. HIV-DNA^−^ sample quality was assessed according to the following parameters: (1) the total number of reads sequenced; (2) the percentage of intergenic and intronic reads; (3) the proportion of ribosomal RNA (rRNA) reads; and (4) the exonic fragment count (Supplementary Table 2). Samples that had a paired-end read count of less than 10^6^ and had >35% mapped intergenic reads were excluded. Furthermore, within each participant, HIV-DNA^−^ samples that differed qualitatively from other replicates by having lower exonic reads or higher rRNA content were removed. If all HIV-DNA^−^ samples were removed for a participant, that participant was excluded from further analysis. After the removal of 31 FIND-seq-sorted samples in this curation process, 22 HIV-DNA^+^ and HIV-DNA^−^ samples belonging to participants 2208, 2510 and 3209 remained (Supplementary Table 2).

### Analysis pipeline testing

The transcriptomes of primary cell samples generated by FIND-seq showed high proportions of intronic and intergenic reads (Extended Data Fig. 4). We therefore performed a second, deeper sequencing of libraries from the J-Lat:Raji cell mixing study and tested whether bioinformatics pipelines that address coverage bias and/or genomic DNA contamination might mitigate the effects of these patterns on the gene expression results. In total, we evaluated nine different pipelines using control data from the J-Lat:Raji cell line mixing study. The details of each pipeline are found below; the default options and parameters were used for all tools unless otherwise noted. Reads were mapped against the GRCh38 (ENSEMBL v.100) reference with coding gene annotations only for all pipelines tested.

#### CLC Genomics Workbench

CLC Genomics Workbench (GWB) v.20 and v.21 (https://digitalinsights.qiagen.com/) were tested using the default settings for mapping and abundance estimation using the RNA-seq analysis tool. For DGE analysis in GWB v.21, the option to filter average expression before FDR correction was selected.

#### 3′ tag counting

Raw reads were preprocessed and mapped using GWB v.21. As in a previous study^70^, reads were mapped to the region within 1,500 bp from the 3′ end of the gene and expression values were calculated in GWB. Analysis of DGE was also performed in GWB.

#### Salmon with positional bias correction

Salmon v.1.3.0 was implemented as it includes an algorithm for transcript expression quantification that incorporates bias modelling to account for position specific and other biases that are commonly seen in RNA-seq data^71^. Read mapping generated from GWB v.20 was used as the input. Post-quantification analysis of DGE was performed using EdgeR (v.3.32.1)^72^ and DESeq2 (v.1.30.1)^73^.

#### SeqMonk DNA contamination correction

We considered that relatively high intergenic read proportions in sorted samples might be due to library incorporation of the genomic DNA retained with each cell during FIND-seq. We therefore used the SeqMonk expression quantification (http://www.bioinformatics.babraham.ac.uk/projects/seqmonk/) pipeline v.1.47.2, which estimates and corrects count data for each transcript using the density of intergenic reads. Read mapping previously processed in GWB v.20 was used as the input. Analysis of DGE was performed in DESeq2. Expression qualification and DGE with or without DNA contamination correction (SeqMonk) was evaluated, and each was tested with or without automatic independent filtering (DESeq2).

### Selection of the analysis pipeline

For each pipeline, transcriptome accuracy was assessed by comparing J-Lat:Raji FIND-seq mixing study DGE results with the DGE detected between J-Lat cells and the unsorted J-Lat:Raji mixture in standard RNA-seq. DEGs were considered as those with an absolute fold change of ≥1.5 and FDR ≤ 0.05. DEGs identified in standard RNA-seq but not in FIND-seq were considered to be false negatives (FN); those identified only after FIND-seq as false positives (FP); and those identified in both FIND-seq and standard RNA-seq as true positives (TP). Based on this, the sensitivity (or recall) as TP/(TP + FN) and positive predictive value (PPV) as TP/(TP + FP) for each analysis process were calculated (Supplementary Table 7).

GWB v.20 and v.21 yielded the highest combination of sensitivity and PPV. Pipelines that corrected for coverage bias and DNA contamination did not increase the sensitivity, and in several cases showed lower PPV. Although GWB v.20 had a higher PPV than GWB v.21, there were developments in the GWB v.21 transcriptome analysis pipeline that were anticipated to reduce noise in primary cell samples. Thus, the pipeline in GWB v.21 was selected for the analysis of participant samples.

### DGE between HIV-DNA^+^ and HIV-DNA^−^ memory CD4 T cells

As described above, transcriptome data from FIND-seq-sorted material contained higher proportions of intronic and intergenic sequences than the standard RNA-seq data. These non-exonic sequences were also abundant in material that was subjected to only the hydrogel encapsulation and cDNA synthesis steps of FIND-seq, consistent with the requisite co-retention of cell genomic DNA with transcriptome material and with efficient nuclear lysis and capture of immature transcripts in our hydrogel-based workflow. Accordingly, after curating the participant samples on the basis of quality, differential expression using only exonic reads was performed (Supplementary Table 3). Using GWB v.21, a combined analysis was performed using the Wald test with Benjamini–Hochberg multiple-testing correction by defining DEGs between HIV-DNA^+^ and HIV-DNA^−^ samples using data from the three participants as biological replicates, while controlling for any interparticipant differences in expression. Moreover, a participant-specific analysis was performed by determining DEGs within each participant separately (Supplementary Table 4). The default settings for all other parameters for the differential expression for RNA-seq tool were used except for Filter on average expression for FDR correction, which was enabled for all analyses. Unless otherwise noted, cut-offs for statistical significance of DEGs were absolute fold change of ≥1.5 and FDR ≤ 0.05.

### Euclidean distance calculation

Pairwise Euclidean distances between the curated samples were calculated using the dist function in R (v.4.1.0) using a matrix of counts per million mapped reads (CPM) gene expression values as input. For each sample of a given HIV DNA status group (that is, HIV-DNA^+^ or HIV-DNA^−^), average intragroup and intergroup distances to all other curated samples were calculated, with values plotted in GraphPad Prism (v.9.3.1). Statistical significance of distance differences between groups was calculated using Mann–Whitney *U*-tests.

### Transcriptomic pathway expression differences between HIV-DNA^+^ and HIV-DNA^−^ cells

Ingenuity Pathway Analysis (Qiagen, summer release 2021) was used to identify enriched biological pathways (Supplementary Table 5) on the basis of DEG lists. For the combined analysis considering samples from different participants as biological replicates, DEGs with an absolute fold change of ≥1.5 and FDR ≤ 0.05 were used. For the participant-specific analysis, DEGs with a fold change of ≥2 and raw *P* ≤ 0.01 were used and pathways regulated in the same direction for all three participants were identified.

The directionality of enrichment of pathways for each analysis was determined from the *z*-score, which is calculated in Ingenuity Pathway Analysis to represent predicted relative pathway activity. The *z*-score for each pathway was calculated using the list of genes annotated to that pathway and meeting criteria for statistically significant differential expression between HIV-DNA^+^ and HIV-DNA^−^ cells. A simplified *z*-score was calculated as follows: *Z* = (*N*^+^ − *N*^–^)/(√*N*), where *N*^+^ and *N*^–^ are those genes of which the direction of regulation is concordant or discordant with predictions from the literature. A positive *z*-score implies activation of a pathway, whereas a negative *z*-score implies inhibition. Statistical significance of the enrichment of a pathway was determined using a right-tailed Fisher’s exact test as described previously^74^. Networks of pathways identified as inhibited across participants and their corresponding genes were plotted using ClusterProfiler (v.4.1.1)^75^.

### WGCNA

Weighted gene co-expression network analysis^76^ was performed in R using the WGCNA package (v.1.70) with a gene expression matrix of CPM values. Genes detected in <2 samples were excluded from analysis. The one-step automatic method was used for network construction and module detection. A soft thresholding power (*β*) of 6 was selected based on approximate scale-free topology using the function pickSoftThreshold. The co-expression network was built with a minimum module size of 30, reassignThreshold of 0 and mergeCutHeight of 0.25. The default values were used for the other parameters. Co-expressed modules of genes that correlated with HIV-DNA^+^ and HIV-DNA^−^ status were identified. Modules that were correlated with the traits with *P* ≤ 0.05 were considered to be significant. GO enrichment analysis for the genes belonging to the two WGCNA modules significantly correlated with cell HIV DNA status was performed using Enrichr (29 March 2021 release)^77,78^. Enrichment analysis was performed using a Fisher’s exact test with Benjamini–Hochberg multiple-testing correction.

### Analysis of HIV reads

To identify sequence reads representing HIV RNA, we created a combined human (GRCh38, ENSEMBL v.100) and HIV (GenBank: KT284371) reference. The HIV sequence for this reference was derived from the clade B representative in the 2016 LANL HIV sequence compendium, with deletions in the LTR regions replaced by the corresponding sequence and annotations from HXB2CG (GenBank: K03455 M38432), and with masking of the *gag* amplicon detected in FIND-seq. Reads were aligned to the combined reference using the Map reads to reference tool with the default settings in GWB (v.21). Counts were obtained for reads extracted from mapping to the combined reference. Mapped reads were visualized using GWB and Integrated Genome Viewer (v.2.11.9).

The frequencies of sequence variants in HIV reads compared to the reference sequence were examined to assess the presence of multiple virus sequences. To do this, a consensus of aligned sequences was generated and reads mapping to the HIV genome were extracted. These reads were then mapped against the consensus reference sequence. The resulting mapping was improved by local realignment in areas containing insertions and deletions (indels). Variants were then identified using the ‘low frequency’ variant caller in GWB v.21 with a minimum coverage of 2, minimum count of 1, inclusion of broken reads and without relative read direction filter applied. The default options for the other parameters were used. The list of variants obtained was manually inspected and filtered to remove (1) those with a frequency above 50% (thus representing the predominant sequence rather than a minor variant) and (2) those with read count = 1 or that represented presumptive technical insertions in homopolymeric regions.

Moreover, the Sequences from HIV Easily Reconstructed (SHIVER)^79^ pipeline (v.1.5.8) was tested to create a hybrid reference from de novo assembled contigs of HIV reads for individual samples and closely matched reference sequences. In brief, reads were mapped to the GRCh38 (ENSEMBL v.100) reference using the Map reads to reference tool in GWB v.21 with stringent settings, with the length fraction and similarity fraction parameters set to 0.8. Unmapped reads were then collected and paired reads among them were processed using the de novo assembly tool in GWB (v.21) with the default settings. We also tested the iterative virus assembler (IVA; v.1.0.11) to perform de novo assembly from the unmapped reads using the default settings, but did not recover HIV contigs using this tool. Contig sequences obtained from GWB (v.21) were exported in fasta format and were processed using the SHIVER pipeline with the default settings. A clade B HIV genome obtained from the 2016 LANL sequence compendium was used as a reference.

### Enrichment analysis of WGCNA modules in defined CD4 T cell subsets

Viably cryopreserved PBMCs from ART-treated people with HIV were thawed and stained for FACS with LIVE/DEAD Aqua stain (Molecular Probes) and the following antibodies (with the indicated dilutions): CXCR5-Alexa Fluor 488 (1:7; BD), CCR5-Cy7PE (1:10; BD), CD27-Cy5PE (1:10; Beckman Coulter), CD45RO-PE-Texas Red (1:12; Beckman Coulter), CD14-PE (1:80; BD), CD11c-PE (1:40; BD), CD3-H7APC (1:5; BD), CCR7-Alexa Fluor 700 (1:8; BD), CD20-APC (1:5; BD), CD56-APC (1:10; BD), T cell receptor gamma delta (TCR-γδ)-APC (1:5; BD), PD1-Brilliant Violet 711 (1:10; BioLegend), CD8-Qdot 655 (1:200; Invitrogen), CD4-Qdot 605 (1:200; Invitrogen), CD57-Qdot 585 (1:50; Invitrogen) and CCR6-Brilliant Violet 421 (1:10; BD). Stained samples were sorted into CD4 T cell subsets using the FACSAria (BD) system by first gating for single cells that were CD3^+^, Aqua^low^ and negative for CD11c, CD14, CD20, CD56 and TCR-γδ. The remaining events that were CD4^+^ and CD8^−^ were then collected as naive (CD27^+^CD45RO^−^) or memory CD4 T cell subsets (see memory subset definitions in Extended Data Table 2). Sorted cell subsets were processed for total RNA extraction and whole-transcriptome sequencing as described previously^63^. The resulting data were processed using the standard pipeline in GWB v.21 using the human reference (GRCh38, ENSEMBL v. 100) with only the coding gene annotations. The resulting CPM values were exported and provided as an input to GSEA (v.4.2.3)^80,81^. Enrichment of module 5 and 28 signatures (separated into genes upregulated and downregulated between HIV-DNA^+^ and HIV-DNA^−^ cells) was identified in transcriptome data from each memory CD4 T cell subset (with data from the naive CD4 T cell subset serving as a reference). GSEA was run using the default settings for all of the parameters.

### Reporting summary

Further information on research design is available in the Nature Portfolio Reporting Summary linked to this article.

## Online content

Any methods, additional references, Nature Portfolio reporting summaries, source data, extended data, supplementary information, acknowledgements, peer review information; details of author contributions and competing interests; and statements of data and code availability are available at 10.1038/s41586-022-05556-6.

### Supplementary information

Reporting Summary

Supplementary Table 1 Clinical characteristics of study participants with HIV. a, Participants studied by FIND-seq. b, Participants studied using FACS analysis.

Supplementary Table 2 Sample curation. a, Sequencing characteristics of all of the samples, including the numbers of sequencing reads, percentages of reads mapped and genomic distributions of mapped reads. b, Sequencing characteristics of FIND-seq-sorted samples that passed a prospective process of curation for library quality (described in the Methods).

Supplementary Table 3 Whole-transcriptome gene expression for curated samples. Raw counts of all genes for all 22 curated samples are shown, determined using GWB v.21 as described in the Methods.

Supplementary Table 4 DGE analysis between HIV-DNA^+^ and HIV-DNA^−^ memory CD4 T cells. The analysis was restricted to protein-coding genes. a, DEGs identified by analysing samples from all of the participants as biological replicates. b, DEGs from participant 2208. c, DEGs from participant 2510. d, DEGs from participant 3209. Statistical significance of differential expression was calculated using Wald Test in GWB v.21.0.3 with Benjamini–Hochberg multiple-testing correction.

Supplementary Table 5 Pathway analysis of DEGs. a, Pathways derived from significant DEGs (defined by absolute fold change ≥ 2, *P* ≤ 0.01) from each participant that were shared and had concordant direction among all three participants. a, Summary of pathway *z*-scores and predicted direction of regulation across participants. b–g, Pathway genes that were differentially expressed in each participant for Gα12/13 signalling (b), NGF signalling (c), Th1 pathway (d), necroptosis signalling (e), dendritic cell maturation (f) and death receptor signalling (g). Significance of differential expression for each gene was calculated using Wald Test in GWB v.21.0.3 with Benjamini–Hochberg multiple-testing correction. Right-tailed Fisher’s exact test was used for statistical testing of pathway enrichment.

Supplementary Table 6 Listing and GO analysis of HIV-DNA^+^ memory CD4 T cell signature genes. Gene modules 5 and 28 were defined and correlated with cell HIV DNA status using WGCNA as described in the Methods. a, Differential expression results of module 5 genes in each participant and for all participants combined. b, GO analysis of module 5 genes. c, Differential expression results of module 28 genes in each participant and for all of the participants combined. d, GO analysis of module 28 genes. Gene co-expression modules were identified using WGCNA v.1.7.0 with weighted Pearson correlation. GO enrichment analysis was performed in Enrichr using Fisher’s exact test with Benjamini–Hochberg multiple-testing correction.

Supplementary Table 7 Selection of analysis pipeline. The sensitivity and positive-predictive value of gene expression quantification and differential expression analysis performed using GWB v.20, GWB v.21 or other pipelines that address coverage bias and/or genomic DNA contamination were tested using J-Lat-Raji cell mixing study data as described in the Methods.

### Source data

Source Data Fig. 2

## Figures and Tables

**Extended Data Table 1:**
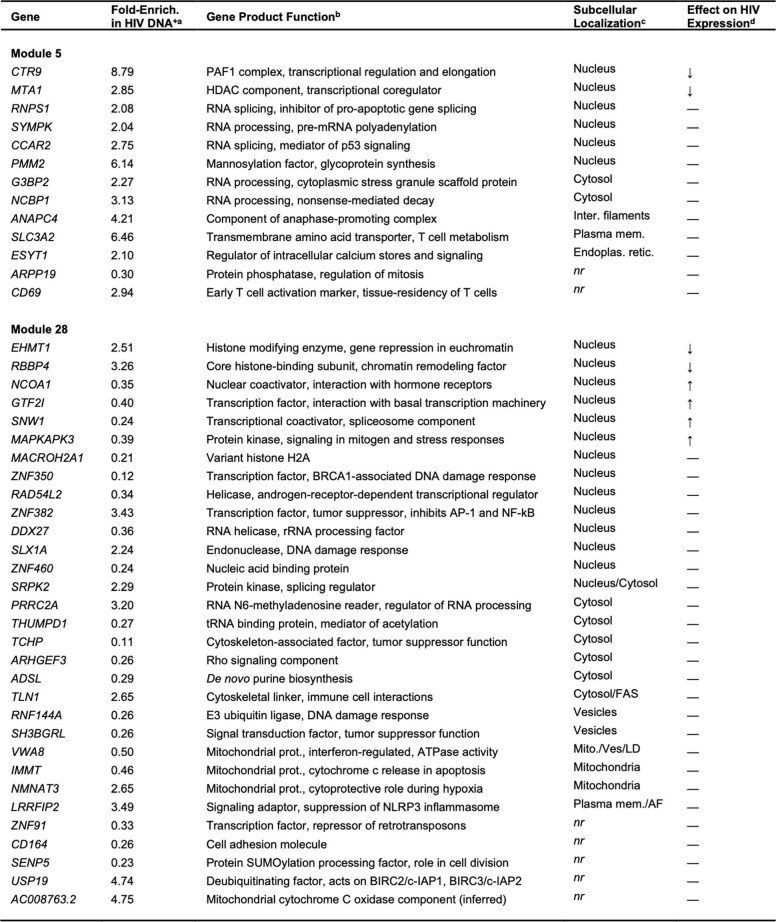
Transcriptomic Signature Genes of HIV DNA^+^ Memory CD4 T Cells under ART

Genes identified in WGCNA modules 5 and 28 that showed a ≥2-fold average expression difference between HIV DNA^+^ and HIV DNA^−^ memory CD4 T cells and a concordant direction across all 3 participants. ^a^Calculated as relative expression level between HIV DNA^+^ and HIV DNA^−^ cells in DGE analysis using GWB v. 21.0.3. ^b^Reported and/or predicted gene product function, curated from https://www.uniprot.org. ^c^Subcellular localization of encoded gene product, curated from https://www.proteinatlas.org. FAS, focal adhesion sites; LD, lipid droplets; AF, actin filaments; *nr*, not reported. ^d^Documented effect of gene product expression on HIV expression level, based on in vitro studies cited in Results.

**Extended Data Table 2:**
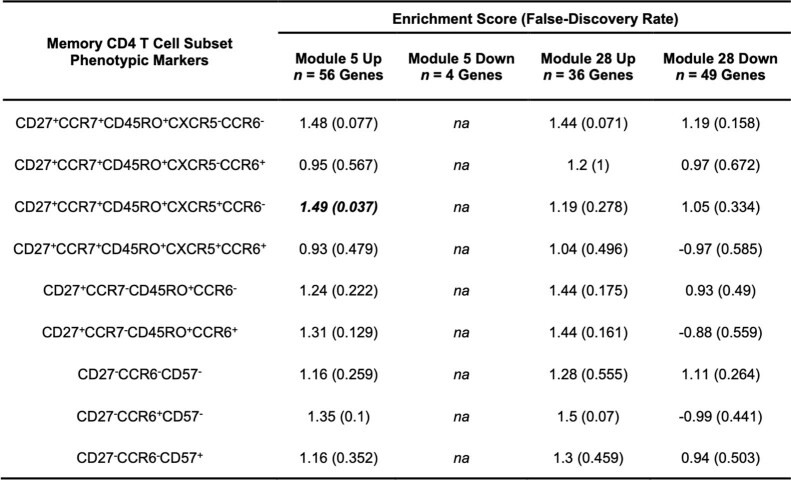
Enrichment of Signature Genes within Sorted Memory CD4 T Cell Subsets from ART-Treated PWH

Naïve and memory CD4 T cell subsets were sorted from PBMC of 9 ART-treated PWH as shown in Extended Data Fig. 6 and were then subjected to standard RNA-seq. Transcriptomic signatures of HIV DNA^+^ memory CD4 T cells defined by WGCNA modules were compared with transcriptomes of memory CD4 T cell subsets using GSEA pre-ranked analysis as described in Methods. Each WGCNA module gene list was separated into two lists, one each for genes that were either higher (up) or lower (down) in HIV DNA^+^ memory CD4 T cells relative to HIV DNA^−^ memory CD4 T cells. A false-discovery rate p ≤0.05 was considered to represent statistically significant enrichment. The enrichment score and false-discovery rate for the CD27^+^CCR7^+^CD45RO^+^CXCR5^+^CCR6^−^ subset are shown in bold italics to indicate statistically significant enrichment for this subset. *na*, not attempted due to gene set size below minimum required for analysis.

## Data Availability

Transcriptome sequencing data from human study participants were deposited with controlled access in the database of Genotypes and Phenotypes (dbGaP; phs003095.v1.p1). Transcriptome sequencing data from cell line experiments were deposited in the NCBI Sequencing Read Archive (SRA; accessions PRJNA819479 and PRJNA893817). Gene sets M3077 and M3076 analysed in Extended Data Fig. 2 are available online (https://www.gsea-msigdb.org/). Source data are provided with this paper.
