# Supplementary material for: HIV silencing and cell survival signatures in infected T cell reservoirs
Source: Nature. 2023 Jan 4;614(7947):318–25. doi: 10.1038/s41586-022-05556-6 (PMC9908556; doi:10.1038/s41586-022-05556-6)
Supplement: Supplementary file 1 [file 41586_2022_5556_MOESM1_ESM.pdf]

nature portfolio

Corresponding author(s): Adam R. Abate, Eli A. Boritz

Last updated by author(s): 10/25/2022

Reporting Summary

Nature Portfolio wishes to improve the reproducibility of the work that we publish. This form provides structure for consistency and transparency in reporting. For further information on Nature Portfolio policies, see our Editorial Policies and the Editorial Policy Checklist.

Statistics

For all statistical analyses, confirm that the following items are present in the figure legend, table legend, main text, or Methods section.

n/a	Confirmed	
☐	☒	The exact sample size (*n*) for each experimental group/condition, given as a discrete number and unit of measurement
☐	☒	A statement on whether measurements were taken from distinct samples or whether the same sample was measured repeatedly
☐	☒	The statistical test(s) used AND whether they are one- or two-sided<br>*Only common tests should be described solely by name; describe more complex techniques in the Methods section.*
☒	☐	A description of all covariates tested
☐	☒	A description of any assumptions or corrections, such as tests of normality and adjustment for multiple comparisons
☐	☒	A full description of the statistical parameters including central tendency (e.g. means) or other basic estimates (e.g. regression coefficient) AND variation (e.g. standard deviation) or associated estimates of uncertainty (e.g. confidence intervals)
☐	☒	For null hypothesis testing, the test statistic (e.g. *F*, *t*, *r*) with confidence intervals, effect sizes, degrees of freedom and *P* value noted<br>*Give P values as exact values whenever suitable.*
☒	☐	For Bayesian analysis, information on the choice of priors and Markov chain Monte Carlo settings
☒	☐	For hierarchical and complex designs, identification of the appropriate level for tests and full reporting of outcomes
☐	☒	Estimates of effect sizes (e.g. Cohen's *d*, Pearson's *r*), indicating how they were calculated

Our web collection on statistics for biologists contains articles on many of the points above.

Software and code

Policy information about availability of computer code

Data collection	Transcriptome sequence data were collected on an Illumina HiSeq 4000 sequencer using HCS 3.4.0 Software.
Data analysis	Sequencing data were pre-processed using bcl2fastq2 script (v. 2.20). Raw reads were further analyzed using various tools in the CLC Genomics Workbench v. 20.0 and 21.0.3 for QC, pre-processing, alignment, gene abundance estimation and differential gene expression steps. Integrated Genome Viewer (IGV) v. 2.11.9 was used for visualizing read mapping. In parallel, Salmon v. 1.3.0 and SeqMonk v. 1.47.2 pipelines were used for aligning and abundance calculation from the transcriptome data followed by differential gene expression using EdgeR (v. 3.32.1) and DESeq2 (v. 1.30.1). For biological pathway and functional enrichment analysis Ingenuity Pathway Analysis (IPA) summer release 2021, clusterProfiler v. 4.1.1 and Enrichr March 29th, 2021 release were used. GraphPad Prism v. 9.3.1 was used for plotting pairwise Euclidean distances between HIV DNA+ and HIV DNA- cells, calculated using 'dist' function in R v. 4.1.0. Weighted Correlation Network Analysis (WGCNA) v. 1.7.0 was used for identifying gene co-expression modules that correlated with cellular HIV infection status. For identifying HIV reads, Sequences from HIV Easily Reconstructed (SHIVER) pipeline v. 1.5.7 and Iterative virus assembler (IVA) v. 1.0.11 were also tested. For examining enrichment of gene sets including module signature identified via WGCNA, we used Gene Set Enrichment Analysis (GSEA) v. 4.2.3.

For manuscripts utilizing custom algorithms or software that are central to the research but not yet described in published literature, software must be made available to editors and reviewers. We strongly encourage code deposition in a community repository (e.g. GitHub). See the Nature Portfolio guidelines for submitting code & software for further information.

Data

Policy information about availability of data

All manuscripts must include a data availability statement. This statement should provide the following information, where applicable:

- Accession codes, unique identifiers, or web links for publicly available datasets
- A description of any restrictions on data availability
- For clinical datasets or third party data, please ensure that the statement adheres to our policy

Transcriptome sequence data from human study participants were deposited with controlled access in the database of Genotypes and Phenotypes (dbGaP; accession phs003095.v1.p1, public study page at https://www.ncbi.nlm.nih.gov/projects/gap/cgi-bin/study.cgi?study_id=phs003095.v1.p1). Transcriptome sequence data from cell line experiments were deposited in the NCBI short read archive (SRA; accessions PRJNA819479 and PRJNA893817). Gene sets M3077 and M3076 analyzed in Extended Data Fig. 2 are available from https://www.gsea-msigdb.org/.

Field-specific reporting

Please select the one below that is the best fit for your research. If you are not sure, read the appropriate sections before making your selection.

☒ Life sciences ☐ Behavioural & social sciences ☐ Ecological, evolutionary & environmental sciences

For a reference copy of the document with all sections, see nature.com/documents/nr-reporting-summary-flat.pdf

Life sciences study design

All studies must disclose on these points even when the disclosure is negative.

Sample size	Samples from 6 people with HIV were selected for this exploratory study without formal sample size calculations. This sample size was anticipated to provide data sufficient for transcriptome sequence analysis using appropriate computational tools.
Data exclusions	Whole transcriptome sequence data from individual samples were excluded from final analyses based on evidence of poor sequencing library quality that could lead to non-representative findings. This was assessed using pre-determined metrics that are described in detail in the manuscript Methods section. Memory CD4 T cells from a sixth person with HIV were also studied, but were excluded upstream of the transcriptome analysis stage based on evidence of sample contamination during the FIND-Seq process.
Replication	FIND-Seq with whole transcriptome sequencing of HIV DNA+ and HIV DNA- memory CD4 T cells was performed once for each of 5 people with HIV, generating up to three biologically independent sorted aliquots of HIV DNA+ cells and up to six biologically independent sorted aliquots of HIV DNA- cells per person. Each sorted aliquot was sequenced and analyzed once.
Randomization	Not applicable - only one grouping of study participants.
Blinding	Not applicable - only one grouping of study participants.

Reporting for specific materials, systems and methods

We require information from authors about some types of materials, experimental systems and methods used in many studies. Here, indicate whether each material, system or method listed is relevant to your study. If you are not sure if a list item applies to your research, read the appropriate section before selecting a response.

Materials & experimental systems

n/a	Involved in the study	
☐	☒	Antibodies
☐	☒	Eukaryotic cell lines
☒	☐	Palaeontology and archaeology
☒	☐	Animals and other organisms
☐	☒	Human research participants
☐	☒	Clinical data
☒	☐	Dual use research of concern

Methods

n/a	Involved in the study	
☒	☐	ChIP-seq
☒	☐	Flow cytometry
☒	☐	MRI-based neuroimaging

Antibodies

Antibodies used	CXCR5-Ax488, BD, cat 558112, clone RF8B2; CCR5-Cy7PE, BD, cat 557752, clone 2D7/CCR5; CD27-Cy5PE, Beckman Coulter, cat 6607107, clone 1A4CD27; CD45RO-TRPE, Beckman Coulter, cat IM2712U, clone UCHL1; CD14-PE, BD, cat 555398, clone M5E2; CD11c-PE, BD, cat 347637, clone SHCL-3; CD3-H7APC, BD, cat 641406, clone SK7; CCR7-Ax680, BD, cat 150503; TCR-gd - APC, BD, cat 555718, clone B1; CD20-APC, cat 340908, clone L27; CD56-APC, cat 341026, clone NCAM16.2; PD1-BV711, BioLegend, cat 329927,

clone EH12.2H7; CD8-Qdot655, Invitrogen, cat Q10055, clone 3B5; CD4-Qdot605, Invitrogen, cat Q10008, clone S3.5; CD57-Qdot585, Invitrogen, clone TB01; CCR6-BV421, BD, cat 562515, clone 11A9

Validation	Antibodies used for flow cytometry were purchased from commercial suppliers and had been validated for use in human samples and published previously.

Eukaryotic cell lines

Policy information about cell lines

Cell line source(s)	3T3 murine cells - American Type Culture Collection (ATCC). J-Lat full-length cells clone 6.3 - NIH HIV Reagent Program. Raji human B cells - American Type Culture Collection (ATCC).
Authentication	Cell lines were used as supplied by above sources, without additional authentication.
Mycoplasma contamination	Cell lines were not tested for mycoplasma contamination.
Commonly misidentified lines (See ICLAC register)	None.

Human research participants

Policy information about studies involving human research participants

Population characteristics	People living with HIV who were >18 years of age, had initiated antiretroviral therapy (ART) during the chronic phase of infection, and had been on ART with suppressed HIV plasma viremia for at least 1 year at the time of study were included. Participants were not selected based on age, gender, or other demographic or clinical parameters. Demographic and clinical laboratory information on study participants is shown in Supplementary Table 1.
Recruitment	Participants were recruited at San Francisco General Hospital to the SCOPE sample collection and cohort study, under an IRB-approved protocol. Investigators conducting the SCOPE study (R.H., S.G.D.) provided samples to the rest of the study team in de-identified fashion.
Ethics oversight	The SCOPE study was IRB-approved at the University of California, San Francisco.

Note that full information on the approval of the study protocol must also be provided in the manuscript.

Clinical data

Policy information about clinical studies

All manuscripts should comply with the ICMJE guidelines for publication of clinical research and a completed CONSORT checklist must be included with all submissions.

Clinical trial registration	NCT00187512
Study protocol	Clinical study record available at https://clinicaltrials.gov/ct2/show/NCT00187512?term=NCT00187512&draw=2&rank=1
Data collection	Data collection occurred at San Francisco General Hospital, San Francisco, CA.
Outcomes	SCOPE is an observational cohort study in which participants receive standard-of-care therapy for their HIV infection. Our exploratory whole transcriptome sequencing study performed within SCOPE included a small number of participants and was not powered to uncover predictors of clinical outcome. Whole transcriptome sequencing results from our study were not cross-referenced with any outcomes or other data collected from study participants subsequent to acquisition of PBMC samples used in sequencing experiments.
